# SB203580, a p38MAPK inhibitor, attenuates olfactory dysfunction by inhibiting OSN apoptosis in AR mice (activation and involvement of the p38 mitogen‐activated protein kinase in olfactory sensory neuronal apoptosis of OVA‐induced allergic rhinitis)

**DOI:** 10.1002/brb3.1295

**Published:** 2019-04-30

**Authors:** Xian Gao, Na Li, Jisheng Zhang

**Affiliations:** ^1^ Department of Otolaryngology‐Head and Neck Surgery The Affiliated Qingdao Municipal Hospital of Qingdao University Qingdao Shandong China; ^2^ Department of Otolaryngology‐Head and Neck Surgery The Affiliated Hospital of Qingdao University Qingdao Shandong China

**Keywords:** allergic rhinitis (AR), apoptosis, mouse model, olfaction dysfunction, olfactory sensory neuron (OSN), p38 mitogen‐activated protein kinase (p38MAPK)

## Abstract

**Objective:**

This study aimed to investigate the effect of the p38 mitogen‐activated protein kinase (p38MAPK) signaling pathway on olfactory mucosa function and apoptosis of olfactory sensory neurons (OSNs) in an allergic rhinitis (AR) mouse model.

**Method:**

Fifty‐five BALB/c mice were used to establish AR models by ovalbumin, and their olfactory function was confirmed by the buried food pellet test. Then, 28 mice with hyposmia were selected. SB203580, a p38MAPK inhibitor, and normal saline (NS) were injected into mice with olfactory defects. The olfactory function, apoptosis of OSNs in olfactory mucosa, and the expression of the olfaction marker protein (OMP), p38MAPK, and p‐p38MAPK were detected after the intervention.

**Result:**

SB203580 treatment significantly upregulated OMP expression and significantly improved the olfactory function of AR mice by reducing the percentage of apoptotic OSNs. In addition, SB203580 attenuated the activation of the p38MAPK signaling pathway.

**Conclusion:**

SB203580 protected olfactory function in an AR mouse model. This protective effect may be associated with the antiapoptotic effects of SB203580 via the p38MAPK signaling pathway.

## 1 INTRODUCTION

Allergic rhinitis (AR) is an allergic disease with increasing morbidity, and olfactory dysfunction is a key symptom of patients with AR. In both seasonal and perennial AR, different proportions and degrees of olfactory dysfunction have been reported. Altogether, the data suggest that, depending on the group studied, olfactory dysfunction could occur with a frequency ranging from 10% to 88% (mode range, 20%–40%). In addition, olfactory function is a key contributor to quality of life (Croy, Nordin, & Hummel, 2014; Stevenson, 2010). At present, the treatment of AR, including glucocorticoids, antihistamines, and antileukotrienes, can improve symptoms such as sneezing, nasal itching, and runny nose but cannot completely improve olfactory function. Moreover, when other symptoms of AR recovered after treatment, there were still some patients whose olfactory dysfunction could not be improved and remained, which has become the most difficult complication of AR to address.

Olfactory dysfunction in the context of AR is typically described in 2 ways, either as conductive or sensorineural (Stuck & Hummel, 2015). Conductive olfactory dysfunction indicates that odors do not reach the olfactory receptor neurons because of a mechanical blockade, such as congested mucosa, polyps, nasal tumors, or increased mucus secretion, creating a mucosal barrier. Previous studies have suggested that mucosal edema or polyps caused by inflammation might block the olfactory cleft, preventing odors from reaching the mucosa of the olfactory area and thus causing olfactory dysfunction. However, some studies have found that olfactory dysfunction can occur even when these obstructive factors are removed. In contrast, sensorineural loss indicates a decrease in olfactory function, in which effective nerve impulses cannot be generated to cause olfactory smell, although odor molecules can reach the olfactory area.

Olfactory function is related to the number of olfactory sensory neurons (OSNs), which are on the surface of the olfactory mucosa. The adult mouse olfactory epithelium (OE) renews itself to maintain a balance between death and generation of OSNs. A decrease in OSNs can directly lead to a decline in olfactory function[n]. OSNs within the peripheral olfactory system are particularly at risk, given their peripheral location and the proximity of their receptive processes (sensory cilia) to the external environment (Hutch & Hegg, 2016; Kim et al., 2015). Because OSNs are the initial site for olfactory signal transduction, their survival is essential for olfactory function (Fanglei, Xiaodong, Weihua, & Tianfei, 2006). Clinically, trauma, aging, and sinusitis are common causes of a decreased sense of smell, with the common pathological process of OSN death caused by apoptosis. Previous studies have suggested that AR‐related inflammatory cytokines, such as those produced by eosinophils and mast cells, may cause structural or functional changes in OSNs on the surface of the olfactory mucosa. However, patients still show irreversible olfactory impairment after treatment with antihistamines, mast cell stabilizers, and glucocorticoids. Related studies have also confirmed that apoptosis does occur on the OE of AR, but the mechanism of OSN apoptosis in the olfactory mucosa of AR is not completely clear.

In studies of AR and allergic asthma, the p38MAPK signaling pathway has been confirmed to participate in and alter the pathological and physiological processes (He, Xie, Ming‐Mei, Hao, & Yang, 2014). The p38MAPK pathway has been confirmed to mediate AR in nasal mucosa inflammation (Xiao et al., 2015). This pathway is the intersection of the cell signaling pathways for the metabolism of regulating cell differentiation and proliferation; at the same time, it can also activate many transcription factors through phosphorylation, which regulates the cytokines involved in cellular responses (Zhang, Yan, Bai, Zhu, & Ma, 2014). Some data have demonstrated that the activation of p38MAPK might be required for olfactory ensheathing cells phagocytosis of neuronal debris, leading to neuron survival and neurite outgrowth.

The purpose of this study was to investigate the effect of the p38MAPK signaling pathway on the apoptosis of OSNs in AR model mice. We expect that this study may help improve the function of the mucous membrane in the olfactory region and mediate OSN apoptosis in the olfactory area, which may effectively treat rhinitis to improve the sense of smell.

## 2 METHODS AND MATERIALS

### 2.1 Mice

Fifty‐five BALB/c mice (female, 6 weeks old, 18–20 g weight) and SPF experimental animals were purchased from Jinan Pengyue Laboratory Animal Co., Ltd. (Jinan, China. RRID:SCR_010607) and housed in the animal center of the Affiliated Hospital of Qingdao University under SPF condition. All procedures and experiments involving animals in this study were performed in accordance with the National Institutes of Health Guide for the Care and Use of Laboratory Animals. The protocol was approved by the Committee on the Ethics of Animal Experiments of the Affiliated Hospital of Qingdao University. All surgery was performed under chloral hydrate anesthesia, and all efforts were made to minimize suffering. One of the mice died in the adaptive feeding period. The other 54 mice were adopted in the experiment.

### 2.2 Mouse model of ovalbumin‐induced AR

Under pathogen‐free conditions, mice were sensitized using ovalbumin (OVA) (Sigma‐Aldrich, St. Louis, MO, USA. RRID:SCR_006144) as follows. OVA (40 μg OVA/200 μl) diluted in sterile normal saline (NS) was administered along with aluminum hydroxide gel (alum adjuvant, 250 mg/10 ml) to unanesthetized animals one time by intraperitoneal injection on days 1, 3, 5, 7, 9, 11, and 13. Then, daily intranasal challenge with OVA, which was diluted with sterile NS (5%, 20 μl of 1 mg OVA/20 ml per mouse), was performed from days 14 to 20. NS was applied to the control group both in the sensitization and the challenge.

On the 21st day, after the last OVA challenge, the symptoms of each mouse (as shown in the Table 1) were observed and recorded. We compared the symptom scores of the two groups of animals at 10 min intervals. Mice with a symptom score above 5 were defined as AR mice.

**Table 1:** Allergic rhinitis symptom scale

Score	Nose scratching	Sneezing times	Clear nasal discharge flow
0	None	None	None
1	Occasionally	1–3	To the prenaris
2	Frequently	4–10	Out of prenaris
3	Ceaselessly	>10	All over the face

### 2.3 Buried food pellet test

The olfactory behavioral tests were carried out with the experimenter blinded in all trials, and the well‐established buried food pellet test (BFPT) was used (Kern, Conley, Haines, & Robinson, 2004; Margolis, 1972). Mice were given a food‐restricted diet (0.2 g chow/mouse/24 hr) for 2 days before testing and during the experimental period but had free access to water. The mice were familiarized with the experimental setting in the enclosure placement for 10 min daily over the 3 days prior to the trials. The olfaction test was conducted twice a day, with an interval over 6 hr. In each trial, one mouse was placed in a test cage alone (45 × 24 × 20 cm) to find a 0.5 g food pellet, which was buried 0.5 cm below the surface of a 3‐cm‐deep layer of mouse bedding material. The location of the pellet was changed every time at random. The time from when the mouse was put into the cage until it grasped the food pellet with its forepaws or teeth was recorded. The mice were allowed to consume the pellet and put back in their cages until the next test. The pads in the test box were replaced after each mouse test. As a comparison, visible food pellets were also tested identically, with the food pellet placed randomly on top of the bedding. This test was carried out on all mice in our preliminary pre‐experiment. All the mice ran to the pellets and consumed them when they were put into the test cage after 2 days of fasting. When the mice were fed sufficient food, they walked around the cage, without any interest in the pellet. Our experimenter did not use any perfumes, lotions, or creams that had odors on the day of the examination.

### 2.4 Measurement of OVA‐specific IgE and IL‐4

Serum levels of IL‐4 and OVA‐specific IgE (Elabsciense, Shanghai, China) were measured by solid‐phase enzyme‐linked immunosorbent assays in accordance with the manufacturer's instructions.

### 2.5 Quantitative real‐time PCR analyses

The olfactory region mucosa was dissected 0.5 hr after the BFPT and immediately frozen in liquid nitrogen for storage at −80°C. Total RNA was extracted with TRIzol reagent (Invitrogen, USA). Reverse transcriptase and oligo dT primer were used to obtain cDNA by 500 ng RNA according to the manufacturer's instructions (TaKaRa, Japan). The mRNA levels of olfaction marker protein (OMP), p38MAPK, and p‐p38MAPK were detected by real‐time PCR using SYBR Green Master Mix. The comparative Ct method (delta delta Ct) was used for relative gene expression analysis. All PCR reactions were performed in duplicate. Primer sequences are shown in the Data S1.

### 2.6 Immunohistological analysis of olfactory mucosa

Heads of mice were excised and fixed in 10% neutral‐buffered formalin for 24 hr at room temperature. After fixation, the heads were decalcified in 8.8% formic acid for 6 days and then embedded in paraffin. Samples were cut into 4 μm cross sections, which were stained with hematoxylin and eosin. The tissue sections were dewaxed in xylene, rehydrated, and rinsed in graded ethanol solutions. Antigen retrieval was performed by heating tissue sections at 100°C for 30 min in citrate (10 mmol/L, pH 6.0) or EDTA (1 mmol/L, pH 9.0) solution when necessary. The sections were then immersed in a 0.3% hydrogen peroxide solution for 30 min to block endogenous peroxidase activity, rinsed in phosphate‐buffered saline for 5 min, and incubated with the primary antibody (Sigma, USA) at 4°C overnight. Negative controls were prepared by replacing the primary antibody with a normal murine IgG antibody. The sections were then incubated with a horseradish peroxidase (HRP)‐labeled antibody directed against a rabbit secondary antibody (Sigma, USA) at room temperature for 30 min. Finally, the signal was developed for visualization with 3,3′‐diamino‐benzidine tetrahydrochloride, and all slides were counterstained with hematoxylin. The color of the antibody staining in the tissue sections was observed by light microscopy.

### 2.7 Western blot analysis

The nuclear protein was prepared using a commercial kit (Thermo Scientific, Rockford, IL, USA). The OE tissue protein was purified. Equal amounts of total protein extracts from cultured cells or tissues were fractionated by 10%–15% sodium dodecyl sulfate polyacrylamide gel electrophoresis and electrically transferred onto polyvinylidene difluoride (PVDF) membranes. Mouse or rabbit primary antibodies and HRP‐conjugated appropriate secondary antibodies were used to detect the designated proteins. The bound secondary antibodies on the PVDF membrane were reacted with ECL detection reagents (Thermo Scientific) and exposed in an Image Quant LAS 4000 mini system (GE Healthcare, Buckinghamshire, UK). The results were normalized to the internal control glyceraldehyde‐3‐phosphate dehydrogenase. Each experimental group was replicated three times. ImageJ analysis software was used to measure the band intensity.

### 2.8 Apoptosis analysis by TUNEL

A TUNEL assay was performed to assess apoptosis of OSNs. The formalin‐fixed paraffin‐embedded OE tissues were cut into 4‐μm sections. Sections were deparaffinized by washing in a graded ethanol series. DNA fragments were determined using an ApopTag in situ apoptosis detection kit (ApopTag Plus Peroxidase; Chemicon; EMD Millipore, Billerica, MA, USA) according to the manufacturer's protocol. For quantification of apoptosis, the stained sections were examined under an optical microscope (magnification x400), and the number of TUNEL‐positive cells was counted in six random microscopic fields and quantified by Image‑Pro Plus 6.0 (Media Cybernetics, Inc., Rockville, MD, USA).

### 2.9 SB203580 intervention in AR mice with hyposmia

AR mice with dysosmia, selected from 54 mice by the BFPT, were randomly divided into three groups. One group was given an intraperitoneal injection of SB20358, the second group was treated with dimethyl sulfoxide (DMSO) (Sigma, USA), and the third group was treated with NS. The BFPTs were used for olfactory detection before and after the intervention. Then, the animals were sacrificed after the last run of the BFPT.

### 2.10 Statistical analysis

Data are presented as the mean ± *SD* unless otherwise indicated. The statistical significance of the difference between the values of the control and treatment groups was determined by Student's *t*‐test using Prism version 5 (GraphPad Software, Inc. RRID:SCR_002798). Values of *p* < 0.05 were considered statistically significant.

## 3 RESULTS

### 3.1 The AR mouse model was successfully established by OVA, and both the BFPT and OMP expression confirmed the olfactory function of the mice

**Figure 1:**
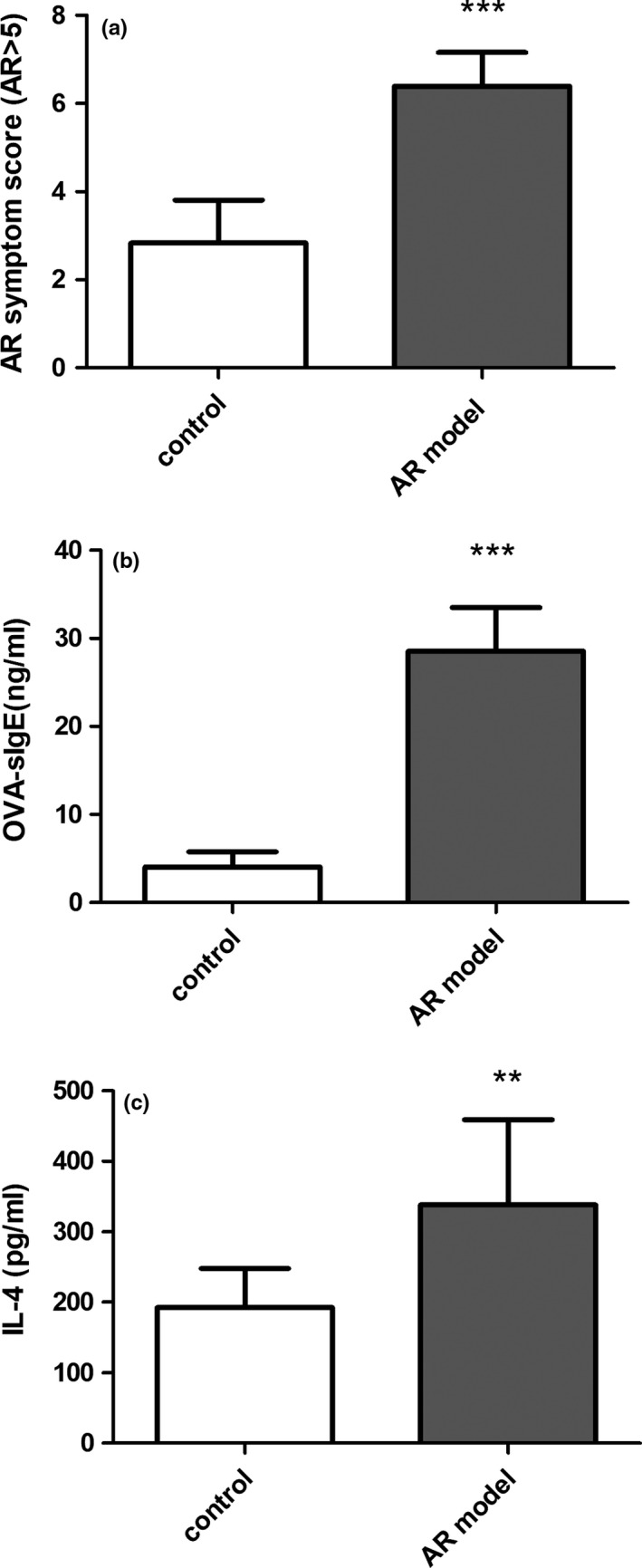
An AR mouse model was successfully established. (a) The symptom scores of mice; (b and c) the levels of OVA‐sIgE and IL‐4 in serum. Data are expressed as the mean ± *SD*. ^∗∗∗^ *p* < 0.001 versus the control group. ^∗∗^ *p* < 0.05 versus the control group. AR, allergic rhinitis; OVA, ovalbumin

BALB/c mice were treated with OVA and confirmed to be a successful model of AR by the symptom score scale (Figure 1a) and the serum levels of OVA‐specific IgE (OVA sIgE) (Figure 1b) and IL‐4 (Figure 1c). The symptom score in the AR group (6.39 ± 0.77) was higher than that of the control group (2.83 ± 0.98). The levels of OVA‐sIgE and IL‐4 in the AR models significantly increased compared with those of the control group, which indicated that the AR mouse model was successfully established.

**Figure 2:**
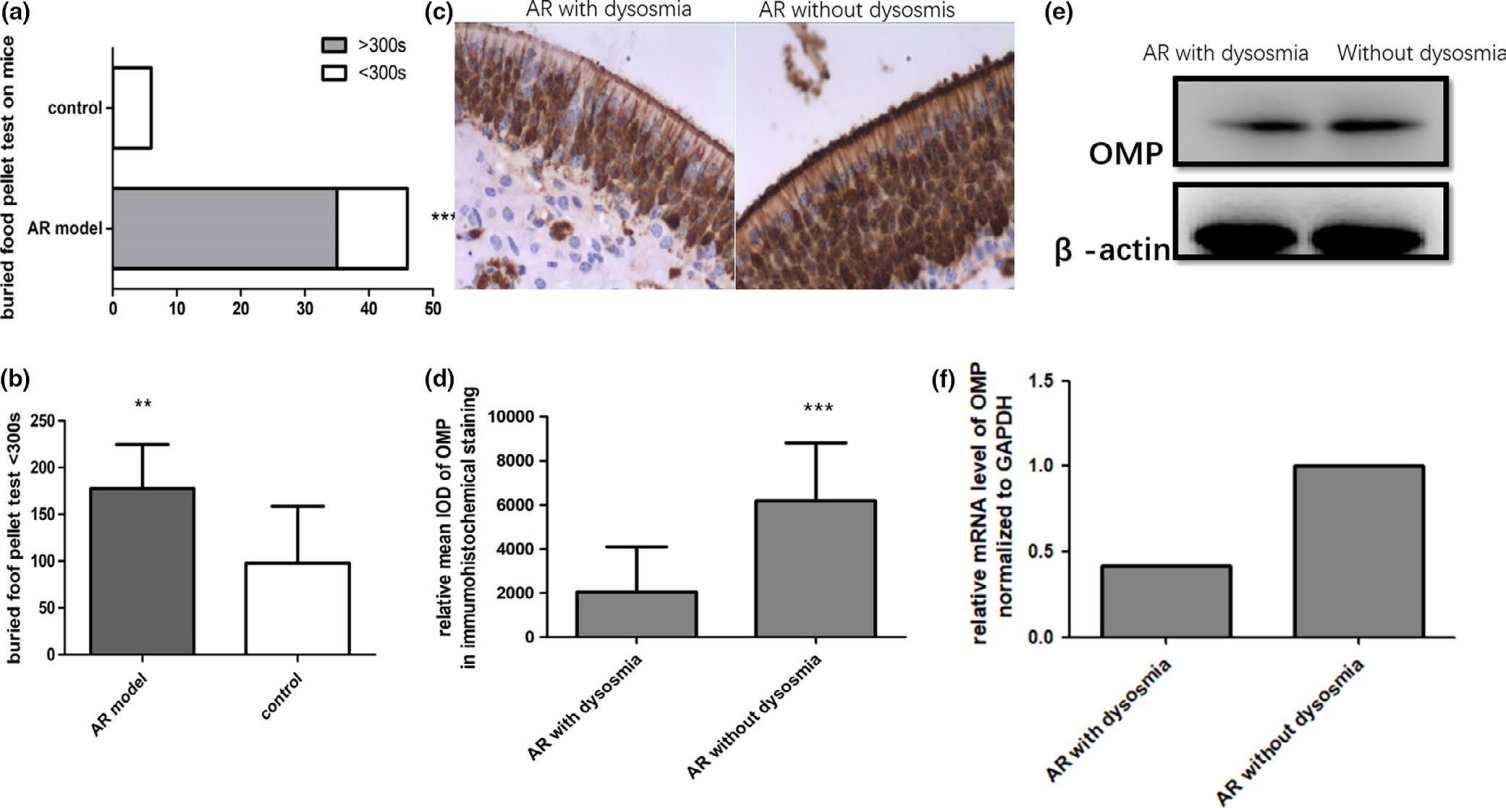
Mice with olfactory dysfunction were selected by the BFPT from AR mice (a and b). OMP expression on olfactory mucosa was downregulated in the dysosmia group by immunohistochemical staining (c and d) and western blotting (e and f). Data are presented as the mean ± *SD*. ^∗∗∗^ *p* < 0.001 versus the control group. ^∗∗^ *p* < 0.05 versus the control group. AR, allergic rhinitis; BFPT, buried food pellet test

The olfactory function of living mice was tested in vivo by assessing olfactory function and OMP expression in the OE. The olfactory function of the AR model decreased compared with that of mice without AR. The BFPT results showed that the foraging time lasted significantly longer in the AR group than the control group. Approximately 74.5% of AR model mice had dysosmia (BFPT > 300 s) (Figure 2a). In addition, AR mice with a time of less than 300 s also had longer foraging times than the control mice (Figure 2b). The expression of OMP on olfactory mucosa declined significantly in the group whose olfactory function decreased. We analyzed the olfactory mucosa of these two groups of mice, those with or without olfaction disorder. Immunohistochemistry, western blot and RT‐PCR analyses revealed that OMP expression was significantly different in the two groups. OMP expression in mice with smell disorders was lower than that in mice without smell disorders (Figure 2c–f). This result was consistent with the OMP expression observed in previous reports as an indicator of olfactory function. Evaluation of OMP expression and the behavioral test confirmed that the AR animals with smell disorders comprised nearly three‐quarters of the AR individuals.

### 3.2 Apoptosis of the OSNs increased in mice with olfactory dysfunction, while the p38MAPK pathway was activated

**Figure 3:**
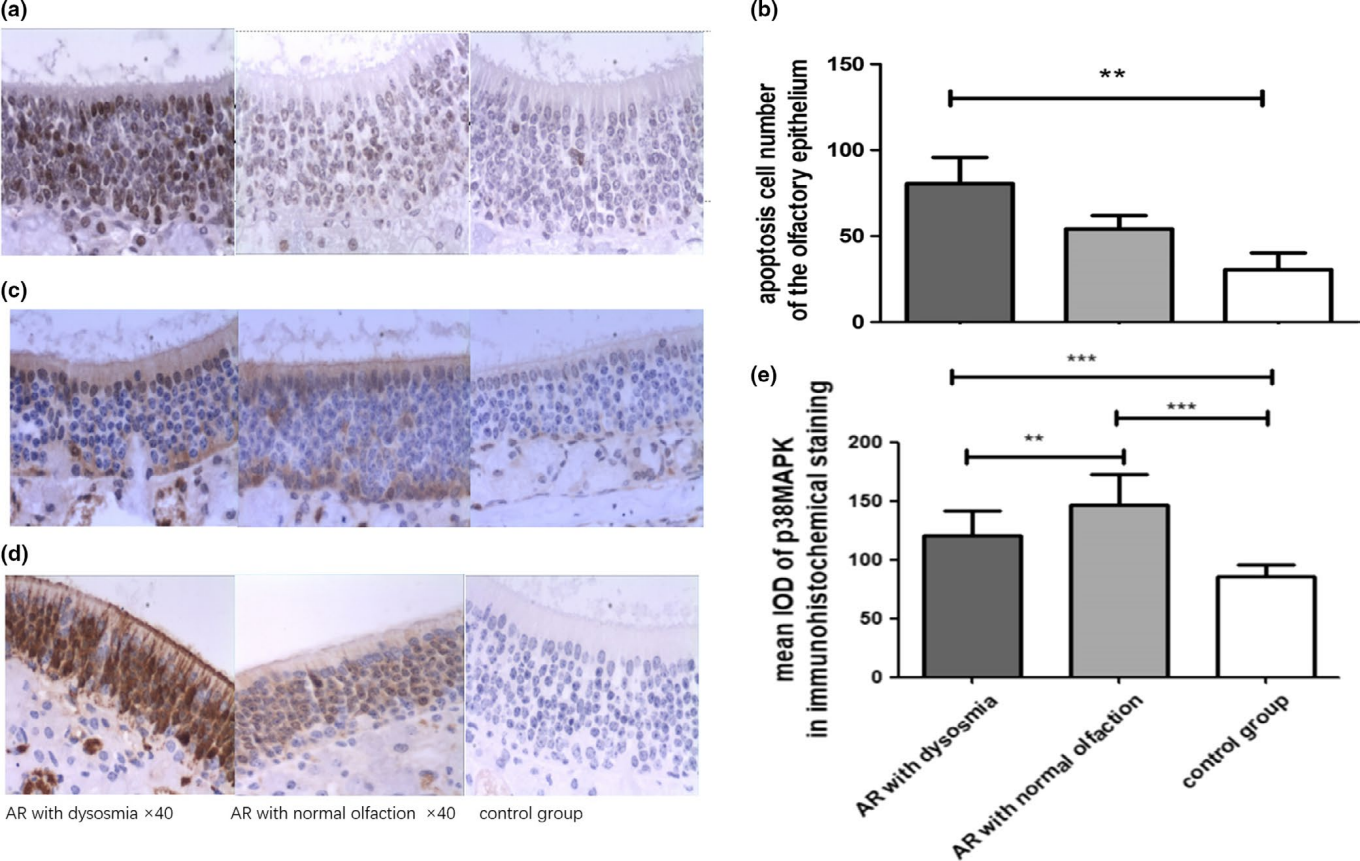
TUNEL test in different groups of apoptotic cells on olfactory epithelium showed that the number of apoptotic cells increased when olfactory function decreased (a and b). The activation of p38MAPK in histological analysis increased in AR mice with dysosmia compared with the control mice (c–e). Data are presented as the mean ± *SD*. ^∗∗∗^ *p* < 0.001 versus the control group. ^∗∗^ *p* < 0.05 versus the control group. AR, allergic rhinitis

The apoptosis of OSNs in the OE of mice with olfactory disorders was detected by TUNEL staining. The number of apoptotic cells in the olfactory area was higher than that in mice with normal olfactory function (Figure 3a,b). As shown by the distribution and morphology of the different cells in the olfactory area, the cells with obvious apoptosis were mostly OSNs. There was strong staining of the nucleus. The expression of OMP on olfactory mucosa significantly declined in those whose olfactory function decreased, whereas the number of apoptotic cells in the olfactory mucosa increased. We collected the olfactory mucosa of the mice with olfactory dysfunction and extracted the total RNA and protein. Western blot, immunohistochemistry and RT‐PCR analyses were used, and the expression of p38MAPK and p‐p38MAPK increased significantly in the dysosmic group (Figure 3c–e). The p38MAPK signal channel was detected during the occurrence and development of AR. This study further suggested that the smell disorders found in AR animals, in which the p38MAPK signal channel may play a new role, are another mechanism that leads to the decline in olfaction.

### 3.3 SB203580, an inhibitor of p38MAPK, improved the olfactory function of AR mice and downregulated apoptosis of OSNs on olfactory mucosa in AR mice

**Figure 4:**
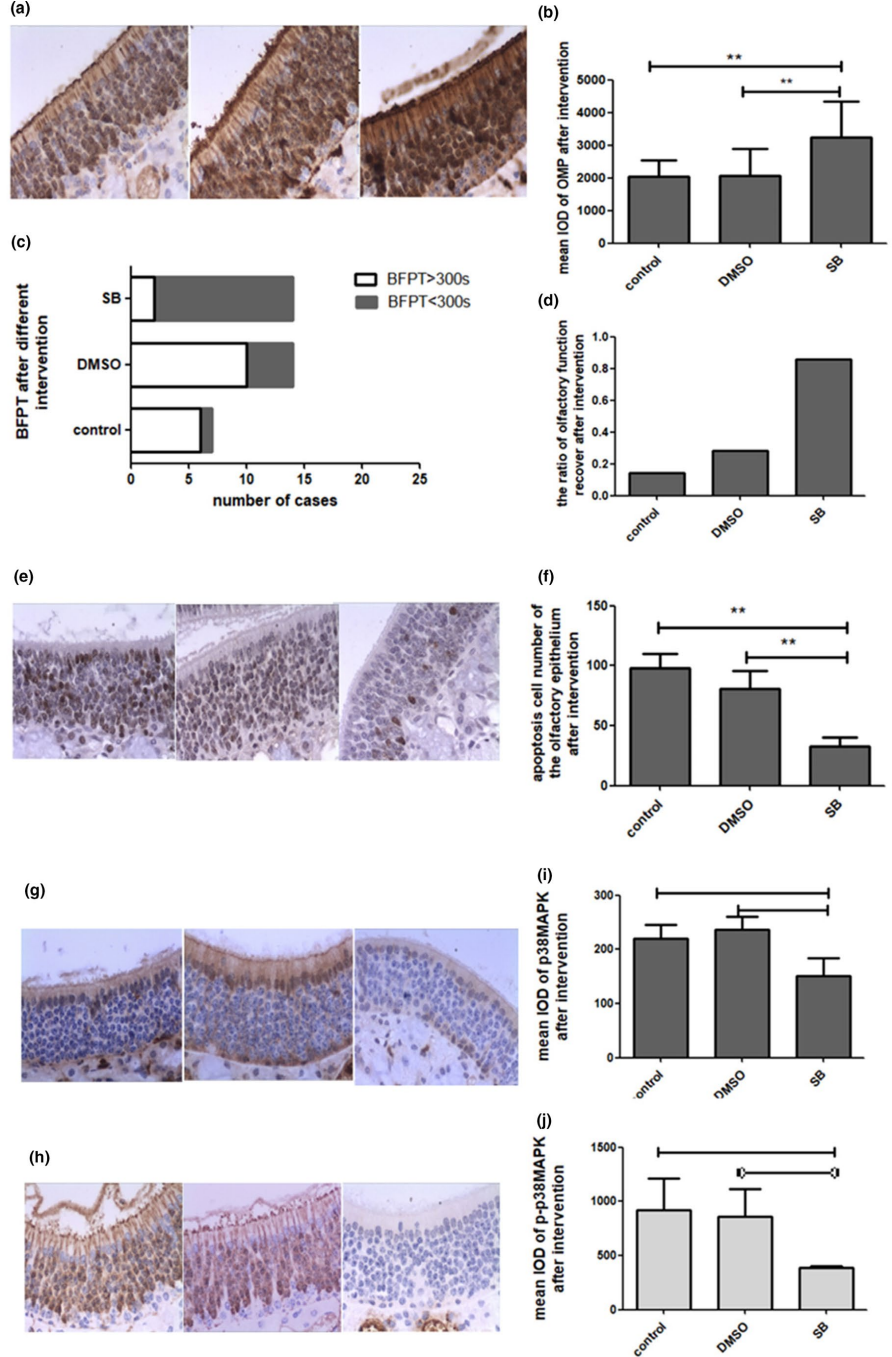
SB203580 improved the expression of OMP (a and b) and improved the olfactory function of dysosmic mice (c and d), whereas the number of apoptotic cells of the olfactory epithelium increased (e and f). SB203580 suppressed the p38MAPK signaling pathway (g, i, h, and j). Data are presented as the mean ± *SD*. ^∗∗∗^ *p* < 0.001 versus the control group. ^∗∗^ *p* < 0.05 versus the control group. OMP, olfaction marker protein

SB203580 is an inhibitor of the p38MAPK signal channel. SB203580 was used to treat mice with olfactory disorder. The results showed that blocking the p38MAPK signal channel not only reduced the symptoms of AR, such as sneezing, nasal itching and nose scratching, but also improved the sense of smell. In the behavioral experiment, approximately 85.71 percent (12/14) of the mice with olfactory dysfunction spent less than 300 s searching for the pellet after the intervention (Figure 4c,d). We found that 85.71% (12/14) of the mice had olfactory function recovery in the SB group. The olfactory function restoration was 35.71% (5/14) in the solvent control group, and the chi‐square test (Pearson) indicated that this difference was significant at *p* = 0.018 (*p* < 0.05); thus, olfactory function was significantly restored after the SB203580 treatment.

We examined the expression of OMP, brown nuclear staining, and the number and layers of OMP on OE after SB203580 treatment and found that these factors were significantly increased, and the staining was darker than that of the dysosmia epithelium in the NS and DMSO groups (Figure 4a,b). P‐p38MAPK and p38MAPK were downregulated after SB intervention (Figure 4g–J). The number of apoptotic cells decreased, and the color of the stained cells was lighter than that of the solvent control, indicating that the apoptosis of OSNs declined after the SB203580 intervention (Figure 4e,f). That is, compared with the control, SB203580 significantly improved olfactory function. At the same time, the expression of OMP before and after intervention also suggested the recovery of olfactory function, as did the observation of apoptotic cells before and after intervention, as SB203580 significantly increased the expression of OMP. These findings further suggested that the number of sensory neurons was positively correlated with olfactory function. Inhibition of the P38MAPK signaling pathway effectively reduced the apoptosis of OSNs, thus improving the olfactory function of mice.

## 4 DISCUSSION

Inhibition of OSN apoptosis may be a feasible treatment (Kern et al., 2004) for olfactory dysfunction (Hutch & Hegg, 2016; Kim et al., 2015). OSNs are the initial site for olfactory signal transduction. Therefore, their survival is essential for olfactory function. In fact, the amount of OSNs on OE is determined by a process of dynamic balance under physiological conditions. OSN apoptosis and basement membrane regeneration lead to a constant balance in the regulation of the final adjustment of olfactory function (Fanglei et al., 2006). Clinically, trauma, aging, and sinusitis are the causes of inhibition of a sense of smell, with the common pathological process of OSN death caused by apoptosis. Olfactory function might be rescued by OSN survival and regeneration through appropriate and effective interventions. In this study, we found that SB203580, an inhibitor of the p38MAPK signaling pathway, effectively inhibited the apoptosis of mature OSNs through the phosphorylated p38MAPK pathway. This finding was consistent with the above conclusions. OMP is a specific marker of the maturity of the olfactory nerve (Margolis, 1972). OMP is expressed only late in cellular development and is widely used as a marker of mature OSN. In our study, OMP was used as a protein marker of mature OSNs. We clearly observed that OMP expression in the OE of normal animals was significantly higher than that in the olfactory dysfunction group. Moreover, after treatment of olfactory dysfunction mice, we found that OMP expression in the OE of mice with improved olfactory function was significantly increased, indicating that the OSN apoptosis level decreased and the number of OSNs increased.

Data from this study support a role for p38MAPK in the apoptosis of OSNs. Our results showed that inhibition of p38MAPK increased OSN expression and significantly improved olfactory function in living mice. These results may provide information that could lead to the development of therapeutic strategies to treat olfactory dysfunction of AR (Benbernou, Esnault, & Galibert, 2013). The roles of particular MAPK pathways in cell death and survival are complex and depend on cell type, magnitude and timing of stimulation. In addition to its role in cell survival, the MAPK pathway is known to influence cell proliferation and differentiation (Frebel & Wiese, 2006). For a long time, three MAPK signal channel pathways were confirmed to participate in the process of cell apoptosis, especially JUK pathways. ERK pathways are mainly linked to cell survival and proliferation (Stanciu, Wang, Kentor, Burke, & Watkins, 2000), whereas JNK and p38 pathways are comprised of stress‐activated protein kinases and implicated in apoptosis (Johnson & Lapadat, 2002; Tournier et al., 2000). Apoptosis mediated by p38MAPK has been confirmed to be one of the major causes of cardiovascular disease and many types of cancers (Maruyama, Sudo, Kasuya, & Shiga, 2000). Phosphorylated p38 was also increased in cultured cerebellar granule cells under glutamate‐induced apoptosis (Chen, Reed, & Lane, 2017; Kawasaki et al., 1997). Depending on the type of stimuli, the cellular response can range from apoptosis to survival. A growing number of studies have found that the effects of different molecules, different signal channels, and different gene regulatory mechanisms in the apoptosis of OSNs are complex and orderly. There must be more than one mechanism to protect OSNs that could act as a survival factor. For example, studies have shown that the bcl‐2/Bax gene in AR is regulated by signaling pathways inside and outside the cell (Benbernou et al., 2013). Interestingly, the mRNA levels of survival genes, such as Bcl‐2, were found to be higher in OSNs treated with odorants than in control cells (data not shown). Experiments have also confirmed that Fas/FasL expression showed significant differences in olfactory mucosa.

One advantage of this study was the analysis of olfactory function in living animals (AR mice) and laboratory tests. Compared with previous studies conducted only in the laboratory, this study evaluated the olfactory function of living animals before and after the intervention so that changes in olfactory function could be observed directly, and the results strongly supported the laboratory test findings. Of course, there are many behavioral methods to examine olfactory function of living animals, such as the BFPT, olfactory analysis instruments, double‐bottle experiments, and others. In our experiment, BFPT was proven to be a convenient and effective method of behavioral examination. In the same prefasting preparation conditions, mice with an impaired sense of smell wandered aimlessly in the box, whereas mice with a normal sense of smell were highly conscious and gradually moved toward the food pellet.

Further improvements could be made to our experiments. Recent literature has shown that apoptosis has two pathways: extracellular pathways (death receptor pathways) and intracellular pathways (telomere pathways), which ultimately result in activation of Caspase‐3 (Hengartner, 2002). Future studies are needed to confirm the specific processes and effectors of the p38MAPK signaling pathway‐mediated regulation of OSN apoptosis. Although the AR animal model adopted at present can reflect the characteristic changes of AR, such as the increase in OVA‐sIgE and changes in Th1/Th2, the course of the disease and the pathological and physiological basis of AR are still different from those of human AR. If the model can be developed based on the actual course of human AR, the decline in the sense of smell of different types of AR will be further studied [3]. Data indicate that the frequency of olfactory dysfunction increases with the duration of the disorder, and its presence seems to increase with the severity of the disease. Of course, more behavioral testing of living animals can be effective in reducing experimental bias. We also expect to find more direct, convenient and stable markers of OSNs that will indicate changes in olfactory function in future studies. In addition, even if the living animals show significant changes in olfactory function, we need to consider whether the changes in the expression of OMP detected in the laboratory are the result of the combined action of various pathological and physiological factors of AR.

## 5 CONCLUSION

In summary, our study investigated the effect of SB203580, a p38MAPK inhibitor, in protecting the olfactory function of AR mice. To our knowledge, this is the first report demonstrating the potential antiapoptotic value of SB203580 in OSNs of AR mice in vivo. Mechanistically, activation of the p38MAPK pathway could suppress olfaction by enhancing the apoptosis of OSNs. Thus, this p38MAPK inhibitor may be a promising candidate that could protect the olfactory function of AR mice from the apoptotic process in OE.

## CONFLICT OF INTEREST

All authors declare no conflict of interests.

## AUTHOR CONTRIBUTIONS

Xian Gao involved in conceptualization, validation, investigation, formal analysis, data curation, writing, and original draft of the manuscript. Na Li involved in conceptualization, methodology, and resources of the manuscript. Jisheng Zhang involved in methodology and validation of the manuscript.

## SIGNIFICANCE STATEMENT

Our results regarding the ability of the MAPK inhibitor, SB203580, to protect olfactory neuronal cells against apoptotic damage that could guide the development of therapeutic agents for allergic rhinitis treatment. Inhibition of OSN apoptosis may be a feasible treatment for olfactory dysfunction. One advantage of this study was the analysis of olfactory function in living animals (AR mice) and laboratory tests. Compared with previous studies conducted only in the laboratory, this study evaluated the olfactory function of living animals before and after the intervention so that changes in olfactory function could be observed directly, and the results strongly supported the laboratory test findings.

## Data Availability

The data that support the findings of this study are available from the corresponding author upon reasonable request.
