# Supplementary material for: SB203580, a p38MAPK inhibitor, attenuates olfactory dysfunction by inhibiting OSN apoptosis in AR mice (activation and involvement of the p38 mitogen‐activated protein kinase in olfactory sensory neuronal apoptosis of OVA‐induced allergic rhinitis)
Source: Brain Behav. 2019 Apr 30;9(6):e01295. doi: 10.1002/brb3.1295 (PMC6577615; doi:10.1002/brb3.1295)
Supplement: Supplementary file 1 [file BRB3-9-e01295-s001.pdf]

SUPPORTING INFORMATION

Article ID: 1295
Article DOI: 10.1002/BRB3.1295

Article title

SB203580, a p38MAPK inhibitor, attenuates olfactory dysfunction by inhibiting OSN apoptosis in AR mice (Activation and involvement of the p38 mitogen-activated protein kinase in olfactory sensory neuronal apoptosis of OVA-induced allergic rhinitis).

Running head

SB203580 attenuates dysosmia by inhibiting OSN apoptosis

Names of authors, their degrees and affiliations

Xian Gao[1,2], Master of Medicine
Na Li[2,] ⊠, M.D., Ph.D.
Jisheng Zhang[2], Ph.D.

1.Department of Otolaryngology-Head and Neck Surgery, The Affiliated Qingdao Municipal Hospital of Qingdao University, Qingdao, Shandong, China
2. Department of Otolaryngology-Head and Neck Surgery, The Affiliated Hospital of Qingdao University, Qingdao, Shandong, China
⊠. Corresponding author.

⊠Corresponding author.
Na Li, Department of Otolaryngology-Head and Neck Surgery, the Affiliated Hospital of Qingdao University, Jiangsu Road No. 16, Qingdao, Shandong 266003, PR China.
Email: dr.lina@163.com

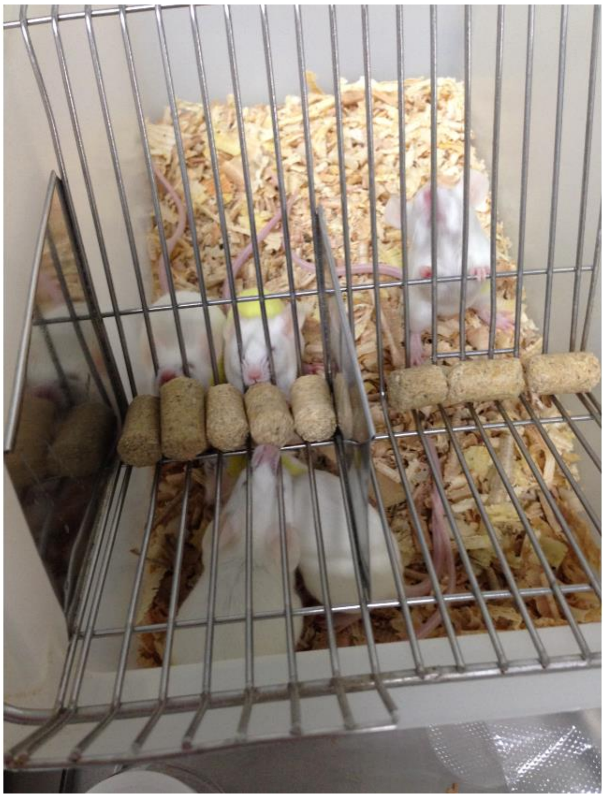

Figure S1 The BALB/c mice in the experiment.

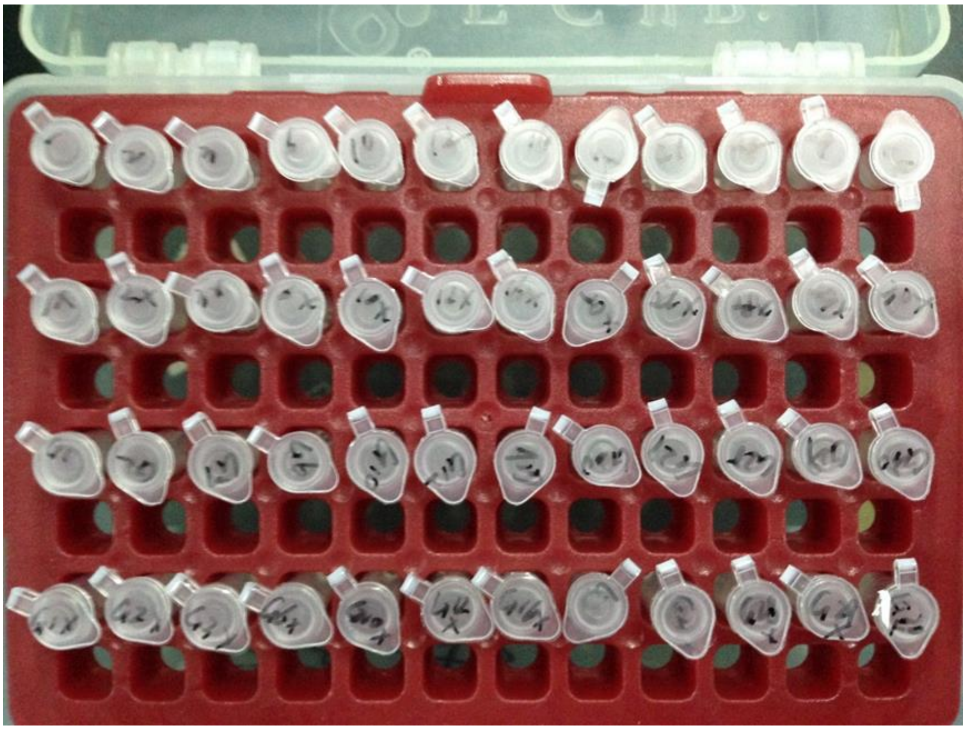

Figure S2 Part of the RNA was extracted from mouse serum

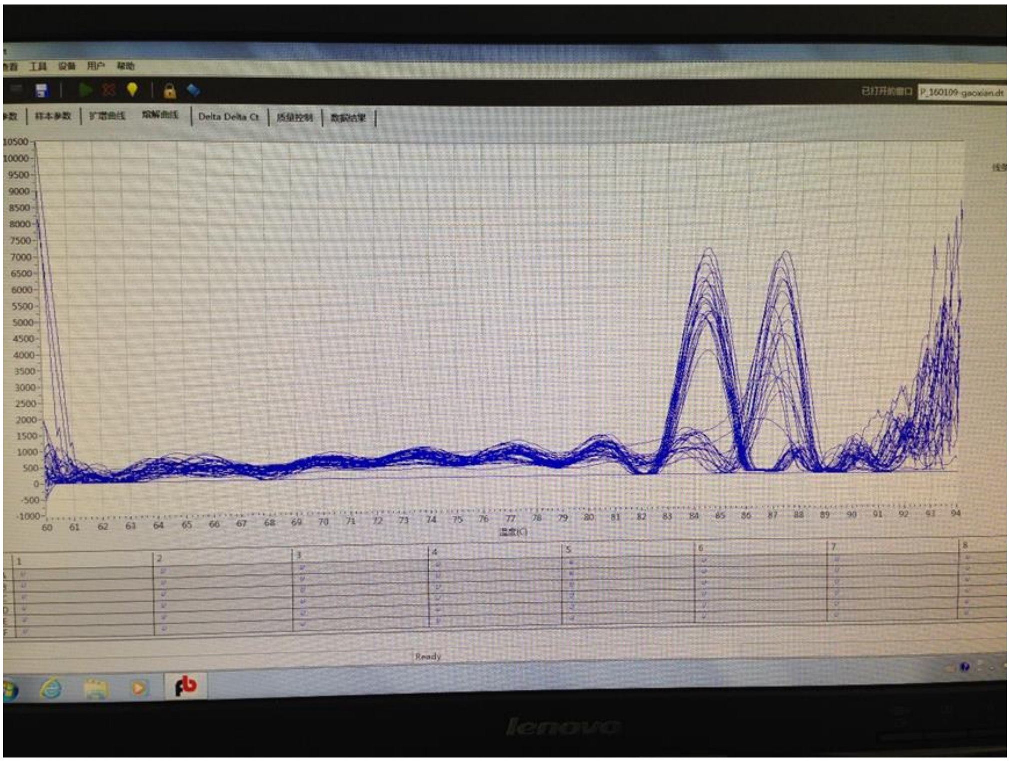

Figure S3 A picture of rt-PCR

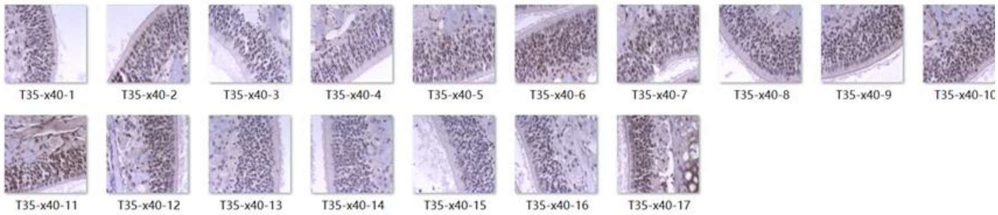

Figure S4 The pictures of OMP high magnification of a mouse olfactory epithelium specimen

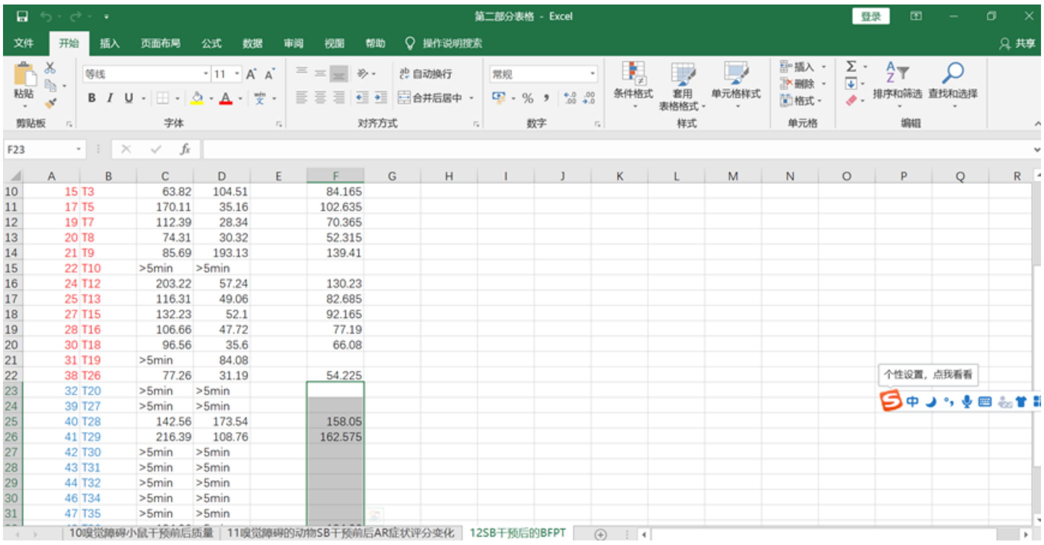

	A	B	C	D	E	F
10	15	T3	63.82	104.51		84.165
11	17	T5	170.11	35.16		102.635
12	19	T7	112.39	28.34		70.365
13	20	T8	74.31	30.32		52.315
14	21	T9	85.69	193.13		139.41
15	22	T10	>5min	>5min		
16	24	T12	203.22	57.24		130.23
17	25	T13	116.31	49.06		82.685
18	27	T15	132.23	52.1		92.165
19	28	T16	106.66	47.72		77.19
20	30	T18	96.56	35.6		66.08
21	31	T19	>5min	84.08		
22	38	T26	77.26	31.19		54.225
23	32	T20	>5min	>5min		
24	39	T27	>5min	>5min		
25	40	T28	142.56	173.54		158.05
26	41	T29	216.39	108.76		162.575
27	42	T30	>5min	>5min		
28	43	T31	>5min	>5min		
29	44	T32	>5min	>5min		
30	46	T34	>5min	>5min		
31	47	T35	>5min	>5min		

Figure S5 Some of the data of BFPT.

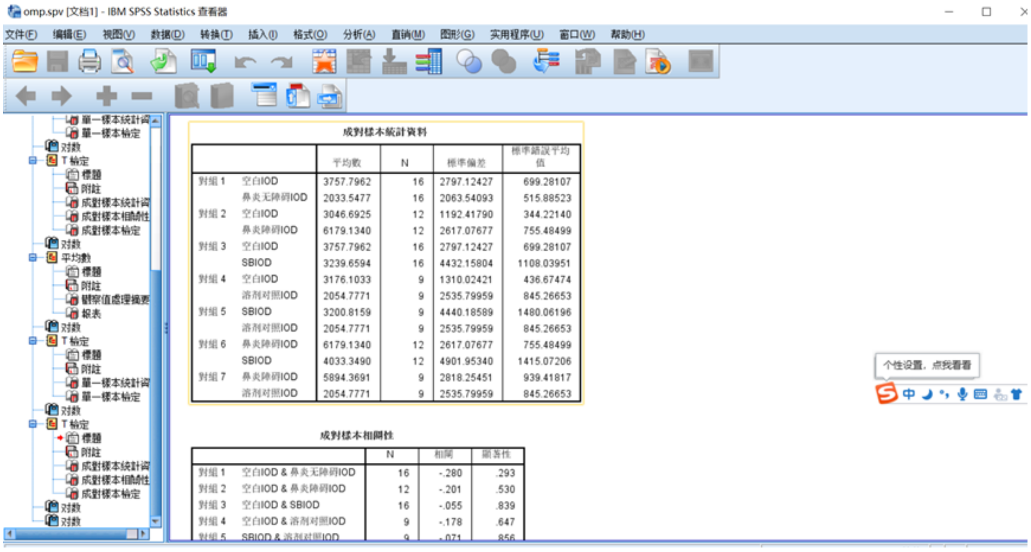

Figure S6 Some of the data of depression of OMP in SPSS.

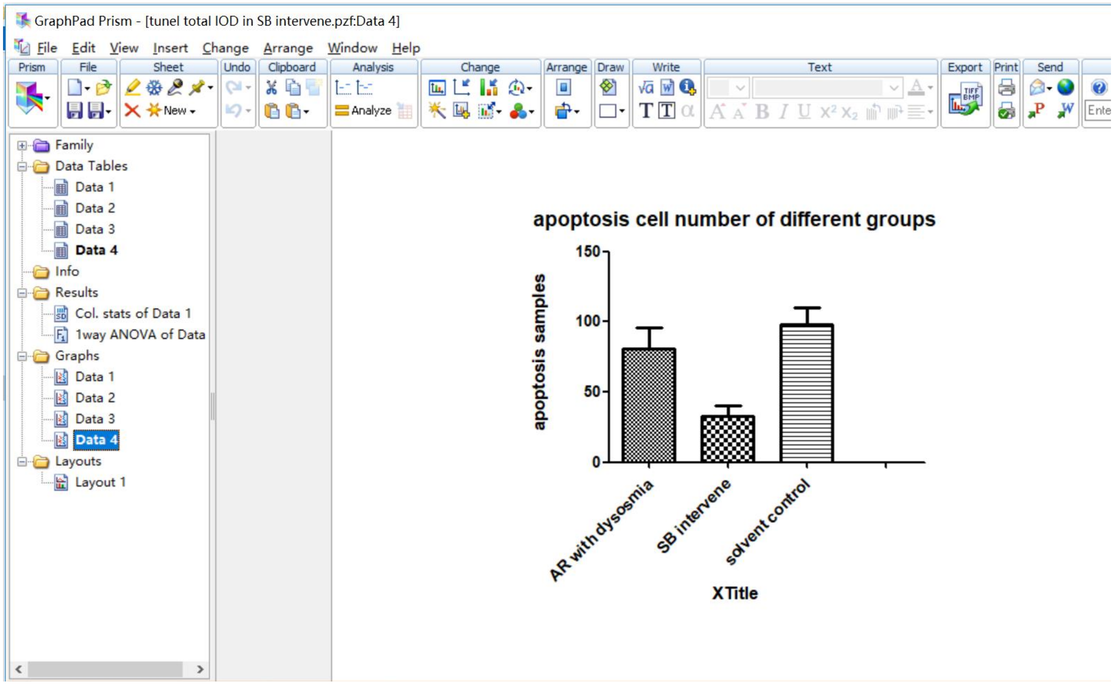

Figure S7 Some of the data of depression of apoptosis cell number in GraphPad Rrism
